# Circulating CD34+ Cell Count is Associated with Extent of Subclinical Atherosclerosis in Asymptomatic Amish Men, Independent of 10-Year Framingham Risk

**DOI:** 10.4137/cmc.s2111

**Published:** 2009-05-27

**Authors:** Lawrence F. Bielak, Richard B. Horenstein, Kathleen A. Ryan, Patrick F. Sheedy, John A. Rumberger, Keith Tanner, Wendy Post, Braxton D. Mitchell, Alan R. Shuldiner, Patricia A. Peyser

**Affiliations:** 1 Department of Epidemiology, University of Michigan, Ann Arbor, Michigan.; 2 Division of Endocrinology, Diabetes, and Nutrition, Department of Medicine, University of Maryland, Baltimore, Maryland.; 3 Department of Diagnostic Radiology, Mayo Clinic and Foundation, Rochester, Minnesota.; 4 Department of Cardiovascular Diseases, Ohio State University, Columbus, Ohio.; 5 Division of Cardiology, Department of Medicine, and the Department of Epidemiology, Johns Hopkins University, Baltimore, Maryland.; 6 Geriatrics Research and Education Clinical Center, Baltimore Veterans Administration Medical Center, Baltimore, MD.

**Keywords:** atherosclerosis, carotid arteries, coronary artery calcification, epidemiology, risk factors

## Abstract

**Background:**

Bone-marrow derived progenitor cells (PCs) may play a role in maintaining vascular health by actively repairing damaged endothelium. The purpose of this study in asymptomatic Old Order Amish men (n = 90) without hypertension or diabetes was to determine if PC count, as determined by CD34+ cell count in peripheral blood, was associated with 10-year risk of cardiovascular disease (CVD) and measures of subclinical atherosclerosis.

**Methods and Results:**

CD34+ cell count by fluorescence-activated cell sorting, coronary artery calcification (CAC) by electron beam computed tomography, and CVD risk factors were obtained. Carotid intimal-medial thickness (CIMT) also was obtained in a subset of 57 men. After adjusting for 10-year CVD risk, CD34+ cell count was significantly associated with CAC quantity (*p* = 0.03) and CIMT (*p* < 0.0001). A 1-unit increase in natural-log transformed CD34+ cell count was associated with an estimated 55.2% decrease (95% CI: −77.8% to −9.3%) in CAC quantity and an estimated 14.3% decrease (95% CI: −20.1% to −8.1%) in CIMT.

**Conclusions:**

Increased CD34+ cell count was associated with a decrease in extent of subclinical atherosclerosis in multiple arterial beds, independent of 10-year CVD risk. Further investigations of associations of CD34+ cell count with subclinical atherosclerosis in asymptomatic individuals could provide mechanistic insights into the atherosclerotic process.

Atherosclerosis is regarded as a localized response to injuries of the endothelial cell layer triggered by factors such as smoking, high blood pressure, and hypercholesterolemia.^1^ Bone-marrow derived progenitor cells (PCs), circulating in the peripheral blood and detectable because they express CD34 on their cell surface, may play a role in repairing injured endothelium.^2^ A subset of these progenitor cells that express both CD34 and markers of endothelial lineage can home to sites of injury and differentiate into endothelial cells to help maintain the integrity of the endothelium.

A decreased number of circulating PCs has been shown to be significantly associated with cardiovascular disease (CVD) events after adjustment for CVD risk factors in selected samples and patients with angiographically documented coronary artery disease.^3^,^4^ Decreased PC counts also have been associated with aging, increased number and level of coronary artery disease risk factors, and increased 10-year risk of coronary artery disease in clinical patients;^4^ however, this finding has not been consistently confirmed in healthy individuals.^5^

The purposes of the current study were to: 1) determine if the number of circulating PCs, as determined by the presence of the CD34 cell surface marker (i.e. CD34+ cell count), was associated with 10-year CVD risk based on the Framingham CVD Risk Prediction Models;^6^ and 2) determine if CD34+ cell counts were associated with the extent of subclinical atherosclerosis, after adjustment for 10-year CVD risk. The study was conducted in asymptomatic Old Order Amish (OOA) men without hypertension or diabetes. Measures of subclinical atherosclerosis included quantity of coronary artery calcification (CAC) and common carotid artery intima-media thickness (CIMT).

## Methods

Participants were recruited through the community-based Amish Family Calcification Study (AFCS). The goals of the AFCS are to examine environmental and genetic risk factors for subclinical atherosclerosis. The Amish tend to have a more homogenous lifestyle compared to other European Americans and are exposed to fewer confounding factors such as use of prescription medications and wide variation in diet, physical activity, education, and socioeconomic status.^7^ Participants were evaluated for traditional CVD risk factors at the Amish Research Clinic in Strasburg, Pennsylvania. An electron beam computed tomography (EBCT) examination of the heart was conducted in Timonium, Maryland and high-resolution B-mode ultrasound of the common carotid arteries was performed at the Amish Research Clinic. The Institutional Review Boards of the participating institutions approved the study. All participants gave informed consent.

CD34+ cell count and CAC measures were obtained in 103 AFCS men who were recruited from the OOA of Lancaster County, Pennsylvania between March 2002 and November 2005. Four men >74 years of age were excluded because the Framingham Risk Equations were developed in asymptomatic individuals who were ages 30 to 74 years of age at baseline. Six men with a history of myocardial infarction or coronary surgery were excluded. One man with a history of hypertension and one man with a history of diabetes were excluded. Finally, one man with an extreme CAC quantity was excluded. This man, age 48 years, a non-smoker with cholesterol of 197 mg/dL, had a 10-year CVD risk of 6.6% and a CAC score of 3,007. The final sample consisted of 90 men, ages 31–74 years with measures of CAC and CD34+ cell count. A subset (n = 57) of these men also had CIMT measurements through their participation in another study.^8^ There was not a significant difference in mean age, 10-year CVD risk, or natural log-transformed CAC quantity between men with and without CIMT measures.

### Risk factor assessment

Height and weight were measured and body mass index (BMI; kg/m^2^) was calculated. Standard enzymatic methods were used to measure total cholesterol, high-density lipoprotein (HDL)-cholesterol, and triglycerides after an overnight fast (Quest Diagnostics, Horsham, PA). Low-density lipoprotein (LDL)-cholesterol was calculated with the Friedewald equation. 9 Systolic blood pressure (SBP; mmHg) and diastolic blood pressure (DBP; mmHg) were measured 3 times in the right arm with a standard sphygmomanometer and the average of the second and third measurements was used in these analyses. Self-reported history of physician-diagnosed myocardial infarction, diabetes, or hypertension were also recorded. As well, self-reported history of coronary surgery or tobacco smoking were recorded.

The Framingham CVD Risk Prediction Model for males was used to estimate the 10-year probability of CVD (10-year CVD risk) that includes coronary heart disease, cerebrovascular disease, peripheral vascular disease, and heart failure based on age, SBP, treatment for hypertension, total cholesterol, HDL-cholesterol, current smoking status, and diabetes status.^6^

### CD34+ cell count

CD34+ cells were counted by a method modified from Vasa and colleagues.^10^ A fasting blood draw was collected in four milliliter Vacutainer^®^ tubes containing 5.4 mg of EDTA. The blood was diluted 1:1 with sterile PBS. Peripheral blood mononuclear cells were isolated by centrifugation at 400 × g utilizing LSM^®^ lymphocyte separation media (ICN Biomedical) per the manufacturer’s instructions. The isolated mononuclear cells were washed twice with sterile PBS to remove platelets. After the second wash, the mononuclear cell pellets were diluted with 900 μL of sterile PBS. Twenty microliters of each cell suspension, which had been diluted in 20 ml of isoton, was treated with Beckman Coulter™ ZAP-OGLOBIN™ II red blood cell lytic reagent and the mononuclear cells were counted in a Beckman Coulter™ Particle Counter Z1 per the manufacturer’s instructions. For the study samples and controls, 1 × 10^6^ cells were treated with FcR blocking reagent (Miltenyi Biotech). Labeling of CD34+ cells was accomplished by incubating with a fluorescein isothiocyanate (FITC)-conjugated mouse anti-human CD34 monoclonal antibody (BD Biosciences) for 15 minutes at room temperature in the dark. Isotype controls were prepared with Mouse IgG1-FITC (BD Bioscience) for 15 minutes at room temperature in the dark. Excess primary antibody was removed with a PBS wash. We also attempted labeling FLK1+ cells as described by Laufs and coworkers^11^ by treating with mouse anti-FLK1 IgG monoclonal antibody (Santa Cruz) followed by labeling with a goat anti-mouse IgG (whole molecule) R-Phycoerythrin conjugate (Sigma) for 15 minutes at room temperature in the dark and washing with PBS. However, subsequently we found that the epitope for the FLK1 antibody was intracellular and thus no binding occurred. Results from follow-up experiments to measure CD34+ cell count with and without the anti-FLK1 antibody were identical, and thus only CK34+ cell count is reported. The study samples and controls were fixed with 2% formalin/PBS solution and stored at 4 °C in the dark until they were counted in an Epics Elite ESP cell sorter (Beckman Coulter) at the University of Maryland School of Medicine Flow Cytometry Core Facility. Each analysis was gated over the lymphocyte area and included 100,000 cytometric events. A correction control prepared with anti-CD3-FITC was used to define cell populations or windows, which were positive and negative for fluorescence. CD34+ cells were counted as cells that exhibited fluorescence in the range of the positive anti-CD3-FITC controls. CD34+ cell count was expressed as the percentage of CD34+ cells/10^5^ cytometric events.

### Subclinical atherosclerosis

CAC presence and quantity were measured with EBCT (Imatron Inc., South San Francisco, California) using a standard protocol. All EBCT scans were scored for CAC by the same cardiologist (JAR) and reviewed by the same diagnostic radiologist (PFS). CAC was defined as a hyperattenuating focus ≥1.0 mm^2^ in a coronary artery and having CT number >130 Hounsfield Units. Quantity of CAC was defined as the CAC score in the four epicardial arteries using the method of Agatston and colleagues.^12^

High resolution B-mode ultrasound was used to image the right and left common carotid arteries (CCAs). A single reader measured CIMT between the intima and media-adventitia interfaces of the far wall of the CCAs (the 1 cm segment proximal to the bifurcation) with an automated edge detection system.^13^ The mean CIMT of this 1 cm segment was measured on two separate images of the left and the right CCA at the peak of the R wave on a simultaneous ECG tracing. The mean of these four measurements was used as the CIMT. Inter-scan reproducibility for CIMT was 89% with this software and the inter-reader and intra-reader reproducibility was 97% and 98%, respectively.

### Statistical analyses

All tests were 2-sided and a significance level of *p* = 0.05 was used for all analyses. CD34+ cell count and CIMT were natural-log transformed to reduce skewness (i.e. ln(CD34+) and ln(CIMT), respectively.) CAC score was natural log-transformed after adding one (i.e. ln(CAC score + 1)) to reduce skewness. Means and standard deviations (SD) for continuous variables, and frequencies and percentages for discrete variables were calculated.

Because individuals in the same family participated in the study, analyses were conducted accounting for the correlations among related individuals. These analyses utilized generalized estimating equations (GEE) with an exchangeable working correlation structure in which all pair-wise correlations between participants from the same family were equal.^14^ Although a complex pedigree structure characterized the Amish participants, families were defined on the basis of sibships for this analysis.

GEE models with the identity link function were fit to assess the association between selected CVD risk factors and ln(CD34+). Each risk factor association, except age, was adjusted for age. A GEE model also was fit to assess the association between ln(CD34+) and 10-year Framingham CVD risk. Finally, GEE models were fit to assess the 10-year Framingham CVD risk-adjusted association between ln(CD34+) and each measure of subclinical atherosclerosis (i.e. ln(CAC score + 1) and ln(CIMT)).

Because each of the dependent variables in the GEE models were natural log transformed values, the parameter estimates were exponentiated to estimate the multiplicative difference in the level of the untransformed dependent variable associated with a specified unit increase in the independent variable.

## Results

The 90 men belonged to 77 sibships: 65 singletons; 11 sibships of size 2; and 1 sibship of size 3. Characteristics of study participants are presented in Table 1. Mean (SD) 10-year CVD risk was 8.4% (6.0%). Mean (SD) CD34+ cell count was 0.125% (0.06%). Forty-two percent of participants had detectable CAC, and the mean (SD) CAC score was 93.8 (282.5). Among the subset of 57 participants examined with carotid ultrasound, the mean (SD) CIMT was 0.62 mm (0.13 mm).

### Associations between risk factors and CD34+ cell count

Age was marginally and inversely associated with ln(CD34+) (*p* = 0.07) (Table 2). After adjusting for age, BMI (*p* = 0.005) and smoking (*p* = 0.02) were each significantly and positively associated with ln(CD34+). Neither 10-year CVD risk nor any of the remaining age-adjusted selected risk factors were significantly associated with ln(CD34+) (Table 2).

### Associations between CD34+ and measures of subclinical atherosclerosis

Ln(CD34+) was significantly and inversely associated with ln(CAC+1) (*p* = 0.01). In a multiple variable GEE model, 10-year CVD risk was significantly and positively associated while ln(CD34+) was significantly and inversely associated with ln(CAC + 1) (Table 3). Based on this model, a 1-unit increase in ln(CD34+) was associated with an estimated 55.2% decrease in CAC quantity (95% CI: −77.8% to −9.3%; *p* = 0.03).

Ln(CD34+) was significantly and inversely associated with ln(CIMT) (*p* < 0.0001). In a multiple variable GEE model (Table 3), 10-year CVD risk was significantly and positively associated while ln(CD34+) was significantly and inversely associated with ln(CIMT). Based on this model, a 1-unit increase in ln(CD34+) was associated with a 14.3% decrease in CIMT (95% CI: −20.1% to −8.1%; *p* > 0.0001).

There was no evidence for a significant interaction between 10-year CVD risk and ln(CD34+) in predicting either measure of subclinical atherosclerosis (data not shown).

## Discussion

Earlier insights into the role of PCs in atherosclerosis were obtained from experiments in atherosclerosis-prone apolipoprotein E (ApoE^−/−^) deficient mice. Periodic injection of bone marrow-derived cells into ApoE^−/−^ mice maintained on high-fat diets significantly reduced the atherosclerotic burden in these animals compared to ApoE^−/−^ mice who received sham injections.^15^ The injection of bone marrow-derived cells appeared to accelerate endothelial regeneration. Importantly, the infusion of bone marrow-derived cells from young donors (either ApoE^−/−^ or wild type) produced a much stronger effect than infusing cells from aged, atherosclerotic donors (either ApoE^−/−^ or wild type).^15^ These results suggested that older animals may be more prone to atherosclerosis due to impaired PC number or function.

Further insight into the role of progenitor cells in atherosclerosis in humans has focused on the use of cells that label both with CD34+, a marker of hematopoetic stem cells, and with KDR+, a marker of endothelial cell fate. In a study of 45 patients with angiographically documented coronary artery disease and 15 healthy volunteers, Vasa et al. found an inverse correlation between CD34+/KDR+ cell count and coronary artery disease risk factors.^10^ In another study of 33 patients with acute coronary syndromes, 44 patients with stable coronary artery disease, and 43 control subjects, the investigators also found an inverse correlation between circulating CD34+/KDR+ cell count and age, hypertension, smoking, and family history of coronary artery disease.^3^ Conversely, Heiss et al. did not find a significant difference in mean CD34+/KDR+ cells between younger (mean ± SD age of 25.0 ± 1.0 years) and older (mean ± SD age of 61.0 ± 2.0 years) healthy individuals.^16^

In our study, we found, after adjusting for 10-year CVD risk, a significant and inverse association between CD34+ cells and measures of subclinical atherosclerosis that are strong predictors of CVD risk. Additionally, we found a positive association between smoking and CD34+ cell counts. This apparently paradoxical positive association also was reported by Werner et al.^4^ in 519 patients with angiographically confirmed coronary artery disease and by Kunz et al.^17^ in 122 patients undergoing cardiac catheterization. Nicotine has been shown to increase endotelial PC count.^18^,^19^ Furthermore, brief exposure to second hand smoke in healthy non-smokers has been shown to immediately increase CD133+/KDR+ and CD34+/KDR+ counts; however, these cells exhibited a severe functional impairment.^20^ OOA men typically smoke cigars and to our knowledge the effect of cigar smoke on PC count and function has not been investigated.

To further explore the significant association between CD34+ cell counts and measures of subclinical atherosclerosis in the current study, we stratified the study group into men with 10-year CVD risk <10% and men with 10-year CVD risk ≥10%. CD34+ cell count was significantly and inversely associated with CIMT and CAC quantity in each of the risk groups. Also, we included BMI as an additional covariate, along with 10-year CVD risk and CD34+ cell count, in prediction of CIMT and CAC quantity in the entire study group. Because of the association between BMI and ln(CD34+) (Table 2), the strength of the associations between ln(CD34+) and the subclinical measures of atherosclerosis were attenuated. The inferences from these models that included BMI as a covariate, however, were similar to the models presented in Table 3.

As reviewed by Saremi and Arora,^21^ quantity of CAC in symptomatic and asymptomatic adults predicts risk for future clinical events.^22^–^24^ In a population-based study of 4,613 participants, CAC score predicted CVD events independently of traditional risk factors and C-reactive protein (*p* = 0.004), was superior to Framingham risk score in predicting events, and enhanced stratification of those falling into the Framingham risk categories of low, intermediate, and high risk (*p* < 0.0001).^24^ In the Atherosclerosis Risk in Communities Study, CIMT was a significant predictor of future coronary heart disease events after adjustment for traditional risk factors.^25^

### Limitations

Our study was conducted in asymptomatic OOA men with a relatively homogeneous social and cultural lifestyle. This limits our ability to generalize our findings to other European American and ethnic populations with more diverse ways of life. We also can not generalize to women. In a previous study, there were significant differences between OOA and other non-Hispanic white Americans in the distribution of many CVD risk factors.^7^ Additionally, the power to detect associations between CD34+ cell count, 10-year CVD risk, and measures of subclinical atherosclerosis in this relatively small sample of men was modest.

There remains controversy as to the appropriate method for defining the population of circulating cells, which constitute endothelial PCs, as these cells express different surface markers depending on their level of differentiation.^26^ Drawing from the method used by Hill et al. and Werner et al. we counted as PCs those cells that labeled with CD34, a marker of hematopoetic and endothelial lineage as well as proliferative capacity.^2^,^4^ We did not, however, measure VEGFR2+ cells. VEGFR2+ cells are thought to represent a subpopulation of CD34+ cells that have further differentiated toward an endothelial cell lineage. Further work will be required to define the subpopulation (if any) of CD34+ cells with the largest impact on endothelial repair and/or atherosclerotic burden.

The CD34+ cell counts we observed are difficult to compare with other studies as there are few published papers that report results for CD34+ cells alone and our study group was restricted to asymptomatic men. In one paper that parallels our approach to measuring CD34+ cells, Fadini et al. report mean CD34+ cell counts of ~0.045% in a population of male and female office workers in Padua, Italy and marginal evidence for an association between CD34+ cell count and CIMT.^27^ Further studies are required to define the distribution of CD34+ cell counts in various populations. The biology of PCs is complex. It was not possible with our cross-sectional study design to provide further insight into pathophysiological mechanisms that underlie the associations found in this study.

## Conclusion

Our findings suggest that CD34+ cell counts may represent a subtle way of assessing subclinical atherosclerotic risk that is not apparent using global risk measures in healthy men. The complex relationships between risk factors, PCs, and subclinical disease underscore the need for additional population-based studies to elucidate biological mechanisms involved in regulation of PCs.

Given the role of PCs in vascular homeostasis, a decline in circulating PC number may represent a biological tipping point, where intrinsic repair mechanisms can no longer effectively maintain vascular health. Individuals with decreased PCs may be at higher risk for events associated with subclinical atherosclerosis in multiple vascular beds and candidates for more aggressive risk factor modification. Further understanding of the role of PCs in the pathogenesis of atherosclerosis may lead in the future to improved outcomes for individuals at risk for CVD.^28^

## Figures and Tables

**Table 1:** Characteristics of 90 asymptomatic men from the Amish family calcification study.

**Characteristic**	**Mean ± SD or %**
Age (years)	48.8 ± 10.0
Body mass index (kg/m^2^)	25.8 ± 2.9
Cholesterol (mmol/L)	5.45 ± 1.00
HDL-cholesterol (mmol/L)	1.45 ± 0.35
LDL-cholesterol (mmol/L)	3.61 ± 0.95
Triglycerides (mmol/L)	0.84 ± 0.42
Systolic BP (mmHg)	115.2 ± 10.6
Diastolic BP (mmHg)	71.1 ± 8.0
Currently smoking	19%
10-year risk of CVD (%)	8.4 ± 6.0
CD34+ cell count (%)	0.125 ± 0.06
Presence of detectable CAC	42%
CAC Score	93.8 ± 282.5
CIMT (mm) (n = 57)	0.62 ± 0.13

Data are mean ± SD unless otherwise indicated.

**Abbreviations:** HDL-cholesterol, high-density lipoprotein-cholesterol; LDL-cholesterol, low-density lipoprotein-cholesterol; BP, blood pressure; CVD, cardiovascular disease; CAC, coronary artery calcification; CIMT, common carotid intimal-medial thickness.

**Table 2:** Risk factor associations with CD34+ cell count in 90 asymptomatic men from the Amish family calcification study.

**Characteristic**	**Unit increase in risk factor**	Percent difference*	**95% Confidence Interval**		***P***
Age	5 years	−4.7	−21.9,	16.2	0.07
Body mass index	1 kg/m^2^	4.8	1.9,	7.8	0.005
Cholesterol	0.25 mmol/L	−0.3	−2.2,	1.6	0.75
HDL-cholesterol	0.125 mmol/L	−0.7	−3.6,	2.4	0.67
LDL-cholesterol	0.25 mmol/L	−0.1	−2.2,	2.0	0.89
Triglycerides	0.125 mmol/L	−0.1	−2.4,	2.1	0.90
Systolic BP	10 mmHg	0.4	−7.4,	8.8	0.92
Diastolic BP	10 mmHg	6.1	−4.6,	17.9	0.27
Currently smoking	Yes versus No	29.5	4.1,	61.0	0.02
10-year CVD risk	2%	−1.9	−5.7,	2.0	0.33

**Abbreviations:** HDL-cholesterol, high-density lipoprotein-cholesterol; LDL-cholesterol, low-density lipoprotein-cholesterol; BP, blood pressure; CVD, cardiovascular disease; CAC, coronary artery calcification.

* Percent increase or decrease in CD34+ cell count associated with specified unit increase in the risk factor. All risk factor associations, except age and 10-year CVD risk, were adjusted for age.

**Table 3:** Risk-adjusted associations between CD34+ cell count and measures of subclinical atherosclerosis.

	**Unit increase in variable**	**Quantity of CAC (n = 90)**			**Common carotid intimal-medial thickness (n = 57)**		
		Percent difference*	**95% Confidence Interval**	***P***	Percent difference*	**95% Confidence Interval**	***P***
10-year CVD Risk	2%	52.2	32.0, 75.5	<0.0001	5.0	3.2, 6.8	<0.0001
ln(CD34+)	1.0	–55.2	−77.8, −9.3	0.03	–14.3	−20.1, −8.1	<0.0001

**Abbreviations:** CAC, coronary artery calcification; CVD, cardiovascular disease; Quantity of CAC, CAC Score; ln(CD34+), natural log-transformed CD34+ cell count.

* Percent increase or decrease in measure of subclinical atherosclerosis associated with specified unit increase in the independent variable.
